# *Epidendrumradicans* Fungal Community during Ex Situ Germination and Isolation of Germination-Enhancing Fungi

**DOI:** 10.3390/microorganisms10091841

**Published:** 2022-09-15

**Authors:** Na Yao, Tao Wang, Xiaolu Cao

**Affiliations:** 1 State Key Laboratory of Tree Genetics and Breeding, Key Laboratory of Tree Breeding and Cultivation of the National Forestry and Grassland Administration, Research Institute of Forestry, Chinese Academy of Forestry, Beijing 100091, China; 2 Beijing Laboratory of Urban and Rural Ecological Environment, Beijing Floriculture Engineering Technology Research Centre, China National Botanical Garden, Beijing 100093, China

**Keywords:** endophytes, mycorrhizae, orchidaceae, *Ceratobasidium*, high-throughput sequencing

## Abstract

Orchids exhibit varying specificities to fungi in different microbial environments. This pilot study investigated the preference of fungal recruitment during symbiotic germination of *Epidendrum radicans* Pav. ex Lindl. Two different orchid substrates were used for ex situ seed baiting: pine bark and rotten oak leaf, with Basidiomycota and Ascomycota as the respective dominant groups. Both substrates promoted seed germination, with a higher protocorm formation rate on pine bark (65.75%). High-throughput sequencing characterized the fungal communities of germinated protocorms. Basidiomycota was the dominant group in protocorms that symbiotically germinated on both substrates. The family-level community structures of endophytic fungi in protocorms that symbiotically germinated on both substrates were close to those of protocorms that germinated in vitro on MS1 medium. For protocorms, the dominant fungal groups recruited from substrates differed at the genus level; from pine bark, they were genera belonging to unclassified Sebacinales (41.34%), *Thanatephorus* (14.48%) and *Fusarium* (7.35%), while, from rotten oak leaf, they were *Rhizoctonia* (49.46%), *Clitopilus* (34.61%), and *Oliveonia* (7.96%). Four fungal isolates were successfully obtained and identified as belonging to the family Tulasnellaceae, genera *Ceratobasidium* and *Peniophora*, which could promote seed germination to the seedling stage. The data indicate that endophytic fungi for *E. radicans* germination on two different substrates are affected at the genus level by the substrate, with a degree of specificity at the family level.

## 1. Introduction

Over hundreds of millions of years, orchids have coevolved with fungi [1]. This coevolution has a range of benefits for orchids and is crucial in seed germination and seedling growth in the natural environment. The seeds of most orchids are tiny and dust-like, lack an endosperm, and contain scant nutritional reserves [2]. For both terrestrial and epiphytic green orchids, seed germination relies on the symbiotic relationships with fungi, particularly orchid mycorrhizal fungi (OMF), to obtain nutrition as minerals, organic nutrients, and carbon energy, and to complete their life cycle [3,4].

The soil or bark in the germination environment is one of the main sources for fungi in orchid germination [5]. The fluctuation of microbial communities in soil may directly affect or act synergistically with factors that are intrinsic for recruitment of fungi during germination of plants [6]. Water and nutrients in habitat soil can also affect fungal composition and orchid distribution [7,8]. Additionally, endophytic or epiphytic fungi of seeds can affect seed germination in rice [9] and oak [10]. More attention has been paid to the composition of endophytic fungal community of roots [11]. However, there are few reports on the community structure of endophytic or epiphytic fungi of orchid seeds.

Orchid seeds recruit fungi from the environment when they fall onto the soil or the bark surface. For some orchids, OMF can colonize seed embryo cells within a week during in vitro symbiotic germination [12]. Isolation of endophytic fungi from in situ or ex situ seed-baiting protocorms or roots may be an effective way to obtain fungi that enhance germination [13,14,15]. Some baited fungi are critical for seed germination. These include fungi belonging to Tulasnellaceae [16], Ceratobasidiaceae [17], and Sebacinales [18]. However, the degree of seed–fungus specificity of most orchids remains unclear. For the leafless epiphytic orchid *Taeniophyllum glandulosum* and the lithophytic orchid *Aerangis ellisii*, the seeds specifically associate with Ceratobasidiaceae [17,19]. *Gymnadenia conopsea* and *Neottia ovata* strongly differed in terms of space distribution, confirming the low specificity with fungal association [20,21].

High-throughput sequencing has been used in microbiota analysis, more comprehensively revealing the community of plant-related fungi (endophytic, epiphytic, or rhizosphere). This has allowed the detection and identification of many fungi that are beneficial for plants [22,23,24]. Amplicon sequencing has detected changes in fungal richness and composition in many orchid root microbiota communities [25]. In recent years, research on the fungal communities of orchid seeds and protocorms in in situ/ex situ germination has gradually increased. Studies have mainly focused on the specificity of seeds and fungal communities, comparison of protocorm fungal communities among different regions, and dynamic changes of fungal communities during germination [26].

**Figure 1:**
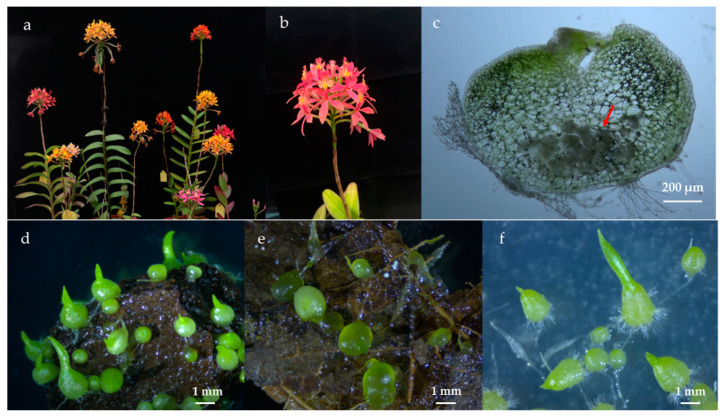
Flowers of *Epidendrum* spp. (**a**) and *E. radicans* (**b**); colonization of fungi in protocorm after symbiotic germination (**c**); protocorms ex situ germinated on pine bark (**d**) and rotten oak leaf (**e**) after 10 weeks of seed baiting; protocorms in vitro germinated on MS1 medium (**f**). The red arrow indicates the intracellular fungal peloton.

*Epidendrum radicans* Pav. ex Lindl. is widely distributed in Central and South America. It readily grows and flowers, and it has a long period of flowering for cut flower production or as an ornamental plant [27] (Figure 1a,b). In the wild, seed germination and plant growth of *Epidendrum* spp. also require mycorrhizal fungi. Roots of *E. rhopalostele* can establish symbiotic relationships with at least two *Tulasnella* spp. [28]. Mycorrhizal fungi belonging to *Tulasnella*, *Ceratobasidium*, and *Sebacina* promote seed germination and seedling development of *Epidendrum* spp. [29,30,31,32].

To investigate the endophytic fungi recruited from different microbial environments and the core fungi during symbiotic germination of *E. radicans*, high-throughput sequencing was used to characterize the fungal community in protocorms of *E. radicans* germinated on different environments (pine bark substrate, rotten oak leaf substrate, and MS1 medium) and substrates. To further study the precise role of these fungi in determining seed germination and the mechanism of fungi promoting germination, the germination-enhancing fungi were isolated from protocorms obtained by ex situ germination. The primary objective of this study was to (1) understand the community structure of endophytic fungi in ex situ germination of *E. radicans* on different substrates, and (2) determine the promoting effect of fungi isolated from protocorms by ex situ seed baiting.

## 2. Materials and methods

### 2.1. Study Materials

*E. radicans* Pav. ex Lindl. was cultivated on pine bark substrate in the greenhouse of Chinese Academy of Forestry, Beijing, China. The plants were cultivated in the greenhouse for 5 years. Their growth was healthy, and they bloomed every year. The fruits were obtained by artificial pollination. Capsules matured approximately 120 days after pollination. Healthy uncracked capsules were harvested in May and October 2021.

The pine bark used for ex situ germination was a commercial New Zealand *Pinus radiata* bark for orchids (Orchiata^TM^). The bark was obtained from the pots of fruiting *E. radicans* in the greenhouse. The rotten oak leaf (*Quercus mongolica*) was a substrate for orchids collected from Changbai Mountain (42°12′ N, 128°05′ E), Jilin Province. All the substrates were collected in May 2021.

### 2.2. Seed Ex Situ Germination and Sampling

The germination containers were 240 mL glass bottles for plant tissue culture. One hundred milliliters of sterile distilled water was added to each container and autoclaved twice at 121 °C for 60 min. The pine bark and rotten oak leaf substrates were separately added to the containers in a clean environment, mixed with the water, and left static for 3 days to allow absorption of sufficient water by each substrate. Excess water was removed, and the saturated substrates were used for ex situ seed germination [33].

The seeds used for ex situ germination were from the mature capsules of 10 individual *E. radicans*. Before sowing, the pericarp was surface-sterilized according to Zi et al. [4]. The capsules were cut with a sterile scalpel on the clean workbench. Mature seeds were removed from the fruits and surface sterilized by a 30 s submergence in 75% ethanol and five 30 s rinses in sterile distilled water.

Approximately 200–300 seeds were sown on the surface of each saturated substrate in each container. For each treatment, 100 bottles were inoculated (10 bottles for each capsule). In vitro seed germination occurred on MS1 medium (see Section 2.5) with 10 bottles for each capsule after sterilization of the seed surface. The culture containers were placed in a growth chamber at 25 ± 2 °C with 16 h of light for 10 weeks.

Seeds that germinated into protocorms were collected from the substrates or medium. The symbiotic germinated protocorms were checked microscopically for mycorrhizal colonization (Figure 1c). Thirty bottles were randomly selected from each treatment (pine bark, rotten oak leaf, and MS1). Approximately 2.0 g of protocorms at stages 4–5 (see Section 2.5) were harvested from the selected bottles, mixed well, and surface-sterilized (30 s submergence in 75% ethanol, 30 s rinse with sterile distilled water, 5 min submergence in 1% sodium hypochlorite, and five 30 s rinses in sterile distilled water). Half of the sterilized protocorms were used for fungus isolation. The other half were stored in sterilized tubes at −80 °C for molecular analysis. Sampling was repeated three times.

### 2.3. Assessment of Fungal Communities in E. radicans Protocorms

To assess endophytic fungal communities of the protocorms from different germination conditions (symbiotic germinated on pine bark and rotten oak leaf, and nonsymbiotic germinated on MS1 medium), sterilized protocorms from the same germination condition were used for sequencing. To obtain the fungal community of substrates, the pine bark and rotten oak leaf substrates in the culture containers were collected and mixed for sequencing. Three replicates of each type of sequencing material were used.

DNA was extracted from 500 mg samples using the QIAamp DNA Stool Mini Kit (QIAGEN, Hilden, Germany) according to the manufacturer’s instructions. The quality and concentration of DNA were detected according to Tian et al. [34]. The internal transcribed spacer (ITS) region of the fungi was amplified using the primers ITS1F (5′–CTTGGTCATTTAGAGGAAGTAA–3′) and ITS2R (5′–GCTGCGTTCTTCATCGATGC–3′) [26] and an ABI GeneAmp^®^ 9700 PCR thermocycler (Applied Biosystems, Waltham, MA, USA). The PCR reactions were conducted using the program according to Chen et al. [26] in triplicate. For each sample, purified amplicons were pooled in equimolar concentrations and paired-end sequenced on an Illumina MiSeq PE300 platform (Illumina, San Diego, CA, USA) according to the standard protocols by Majorbio Bio-Pharm Technology Co. Ltd. (Shanghai, China). The raw reads were deposited into the NCBI Sequence Read Archive Database as accession number PRJNA842498.

The raw ITS region sequencing reads were demultiplexed, quality-filtered using fastp version 0.20.0 [35], and merged using FLASH version 1.2.7 [36], according to the criteria used by Tian et al. [34]. 

Operational taxonomic units (OTUs) with a cutoff of 97% similarity were clustered using UPARSE version 7.1 [37]. Chimeric sequences were identified and removed. The taxonomy of each OTU representative sequence was analyzed using RDP Classifier version 2.2 [38] against the UNITE 8.0 ITS [39] with a confidence threshold of 0.7. Differences were compared according to Student’s *t*-test and Tukey’s test. The analyses and visualization of alpha diversity, principal coordinate analysis (PCoA), and hierarchical clustering were realized using vegan 2.5–6 package in R v3.6.0 [40] and imageGP (http://www.ehbio.com/ImageGP (accessed on 6 August 2022)) [41].

### 2.4. Fungal Isolation and Identification

Surface-sterilized protocorms collected from substrates were used for fungal isolation. The protocorms were sliced into sections and inoculated on one-quarter strength potato dextrose agar (one-quarter strength of PDA from BD Difco with 11.25 g/L agar) medium at 25 ± 2 °C. Five sections were placed in one petri dish for fungal isolation with 20 dishes per treatment. When hyphae growing from the sections exceeded 0.5 cm in length, tips of the hyphae were cut and transferred to new one-quarter strength PDA medium for purification [13]. After repeating this purification step four to five times, purified isolates were obtained. All obtained fungal isolates were identified morphologically by observing and recording the color and surface shape of culture characteristics on PDA medium, as well as the mycelial morphology and spore production under light microscope [30,42]. 

For molecular identification of the isolates, the rDNA region containing the two ITS regions and the 5.8S gene was amplified using the ITS1 and ITS4 primers [43]. PCR was performed as previously described [44]. The PCR products were purified and sequenced at Sangon Biotech Co., Ltd. (Beijing, China) using the same primers. The sequences were BLASTed against the GenBank database of the National Center for Biotechnology Information for identification. The sequences were deposited in the GenBank database (Table 1). The germination-enhancing isolates were maintained at the Research Institute of Forest, Chinese Academy of Forestry.

### 2.5. Testing Fungal Promotion of Seed Germination

All identified fungi were tested for their ability to promote germination of mature seeds of *E. radicans* obtained by artificial pollination between different individuals in greenhouse. After surface sterilization of the seeds as described above, approximately 200–300 seeds were sown on OMA medium (3.0 g of oatmeal (Solarbio, Beijing, China) was added with 800 mL of distilled water, boiled for 30 min, filtered through a 50 mesh sieve to remove large particles, and then fixed to 1000 mL with 6.0 g of agar) in a petri dish. A fungal inoculum was placed in the center of each dish and co-inoculated with seeds. Asymbiotic germination was performed as control on OMA and MS1 (MS medium with one-quarter strength MS macronutrients, 0.4 mg/L 6-benzylaminopurine, 10.0 g/L sucrose, 6.0 g/L agar, pH 6.3) [45]. There were three replicates for each treatment. All replicates were placed in germination chambers at 25 ± 2 °C using alternating 12 h periods of light and dark cycles. Three independent sowing experiments were performed. 

The number of seeds and the status of seed germination in each dish were assessed 30 and 90 days after incubation, according to previously defined stages [46]. The percentages of germinated seeds at each developmental stage were calculated. Seed germination data were recorded as previously described [47]: 0, 1, (2 + 3), and (4 + 5) stages were defined on the basis of the number of ungerminated, germinated (g) seeds, protocorms (p), and seedlings (s). The total number of seeds (t) constituted the seeds with well-developed embryos. Germination rate (G) and protocorm formation rate (P) were calculated 30 days after inoculation. Seedling formation rate (S) was calculated 90 days after inoculation [18]. The following calculations were used:

G = 100 × (g + p)/t.(1)

P = 100 × p/t.(2)

S = 100 × s/t.(3)

Analysis of variance was performed using SPSS 16.0 (SPSS Inc., Chicago, IL, USA). The data were analyzed using one-way analysis of variance (ANOVA) after inverse sine transformation. Statistical significance was set at *p* < 0.05. The means of samples were compared using the least significant difference.

## 3. Results

### 3.1. Efficiency of Ex Situ Germination on Different Substrates

After 10 weeks of symbiotic germination on the substrates, protocorms were harvested at stage 4–5 from the two types of substrates. Both pine bark and rotten oak leaf substrate could promote seed germination. However, protocorm formation rate on pine bark was significantly higher than on rotten oak leaf (65.75% ± 6.73% vs. 44.58% ± 5.12%; *p* < 0.05), with superior protocorm consistency and quality (Figure 1d,e).

### 3.2. Fungal Community Composition

After quality filtering and removal of chimeric and plant reads, 933,732 high-quality ITS sequences were obtained from 15 samples. These included two substrate samples (pine bark and rotten oak leaf) and three protocorm samples (ex situ germinated on pine bark and rotten oak leaf separately, and in vitro germinated on MS1 medium) (Figure 1d–f), with three replicates of each sample. Each sample contained 36,467 (in vitro germinated protocorm) and 152,894 (rotten oak leaf substrate) ITS sequences. A total of 779 OTUs were observed at 97% similarity. All the Shannon curves on the operational taxonomic unit (OTU) level tended to approach the saturation plateau, indicating that the number of sequenced reads in each sample was reasonable (Figure S1). After subsampling according to the sample with the least number of sequences, 715 OTUs were observed at 97% similarity. These were assigned to eight phyla, 153 families, and 265 genera. More OTUs were found in rotten oak leaf substrate (529 OTUs) than in the in vitro germinated protocorm (53 OTUs) (Table S1).

**Figure 2:**
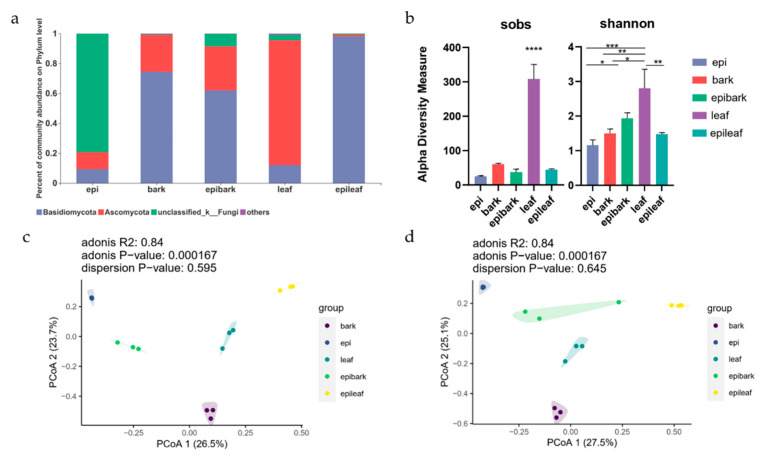
Fungal community compositions of protocorms and substrates at the phylum level (**a**); alpha diversity of fungal OTUs (**b**); principal coordinate analysis (PCoA) plots of fungal community structures at the genus level (**c**) and family level (**d**) based on the Bray-Curtis distance matrix. epi: protocorms in vitro germinated on MS1 medium; bark: pine bark substrate; epibark: protocorms ex situ germinated on pine bark; leaf: rotten oak leaf substrate; epileaf: protocorms ex situ germinated on rotten oak leaf. * *p* < 0.05; ** *p* < 0.01, *** *p* < 0.001; **** *p* < 0.0001.

At the phylum level, the predominant fungi in all samples were mainly from Basidiomycota, Ascomycota, and unclassified fungi. The dominant group in symbiotic germinated protocorms belonged to Basidiomycota (germinated on pine bark: 62.07%; germinated on rotten oak leaf: 98.17%). In in vitro germinated protocorms, except for unclassified fungi (79.40%), the relative abundances of Ascomycota (11.26%) and Basidiomycota (9.34%) were lower than symbiotic germinated protocorms. The dominant groups were Basidiomycota on pine bark (74.40%) and Ascomycota on oak leaf (83.40%). Fungi from rotten oak leaf and protocorms on rotten oak leaf exhibited different patterns in OTU richness at the phylum level. The abundance of Ascomycota was high in rotten oak leaf substrate; in corresponding symbiosis, the abundance decreased significantly from 83.40% to 1.17% (*p* < 0.01). For pine bark and symbiotic protocorms on pine bark, the relative abundance of the dominant phyla did not change significantly (Basidiomycota from 74.40–62.06%; Ascomycota from 24.62–29.28%; both *p* > 0.05) (Figure 2a).

### 3.3. Diversity of Fungal Communities

To evaluate the diversity of fungal community associated with three types of protocorms and two substrates, alpha diversity was calculated across samples. For substrates, the community richness at the OTU level was higher than corresponding symbiotic germinated protocorms, with the highest diversity level in rotten oak leaf (*p* < 0.01). For symbiotic germinated protocorms, there was no significant difference among the OTU numbers (80 OTUs for protocorm on pine bark and 87 OTUs for protocorm on oak leaf) (Figure 2b). The significant difference in community diversity between two substrates did not lead to a significant difference in symbiotic germinated protocorms on the different substrates at the OTU level (Figure 2b).

PCoA plots at the genus level showed that samples generally clustered together on the basis of different substrates, indicating distinct communities. Fungi associated with rotten oak leaf and ex situ germinated protocorms on rotten oak leaf were distinct from other treatments (pine bark substrate, protocorms ex situ germinated on pine bark, and protocorms in vitro germinated on MS1 medium) (Figure 2c). PCoA plots at the family level revealed three types of protocorms clustered together, implying that the community structures of endophytic fungi in protocorms germinated on different microbial environments were close at the family level (Figure 2d). The fungal community of nonsymbiotic germinated protocorms was closest to symbiotic germinated protocorms on bark substrate at both genus and family levels (Figure S5).

### 3.4. Taxon Composition of Fungal Communities in Protocorms

**Figure 3:**
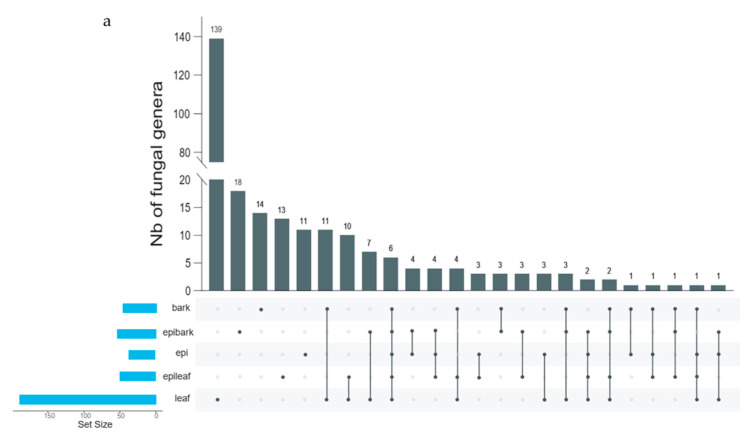

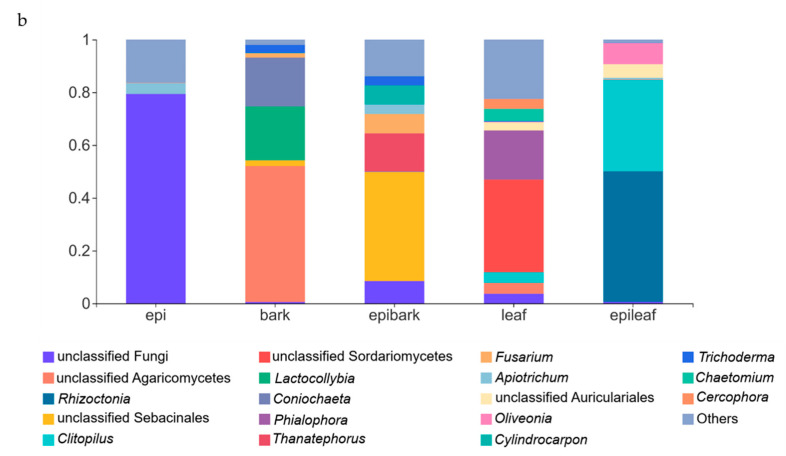
Fungal genera shared among different protocorms and substrates (**a**); fungal community compositions of protocorms and substrates at the genus level (**b**). epi: protocorms in vitro germinated on MS1 medium; bark: pine bark substrate; epibark: protocorms ex situ germinated on pine bark; leaf: rotten oak leaf substrate; epileaf: protocorms ex situ germinated on rotten oak leaf.

In the pine bark treatment, nine genera were shared for symbiotic germinated protocorms and pine bark substrate, which were recruited from pine bark by seeds during symbiotic germination (Figure 3a). In symbiotic germinated protocorms, four of the nine shared genera were dominant genera (unclassified Sebacinales 41.34%, *Fusarium* 7.35%, *Cylindrocarpon* 7.19%, and *Trichoderma* 3.44%). *Thanatephorus* (14.48%) was also one of the dominant groups, which was only detected in symbiotic germinated protocorms on pine bark (Figure 3b).

In the rotten oak leaf treatment, 16 genera were shared by symbiotic germinated protocorms and rotten oak leaf (Figure 3a). Some of these shared genera were also dominant groups in protocorms, such as *Clitopilus* (34.61%), *Oliveonia* (7.96%), and unclassified Auriculariales (5.15%). *Rhizoctonia* (49.46%) as the most abundant group was only detected in symbiotic germinated protocorms on oak leaf (Figure 3b).

In total, 12 genera (two phyla, seven classes, seven orders, and nine families) were shared by all three types of protocorms in seed germination (Figure 3a). The relative abundance of unclassified fungi was the highest (79.06%). Genera including *Apiotrichum* (7.53%), *Cladosporium* (4.23%), *Aspergillus* (2.81%), *Thermomyces* (1.69%), *Fusicolla* (1.30%), *Cutaneotrichosporon* (1.29%), and *Russula* (0.80%) were detected as the fungal core community for all three types of protocorms.

### 3.5. Identification of Isolated Fungi and In Vitro Symbiotic Germination

Fungi that morphologically resembled OMF were isolated from symbiotically germinated protocorms. Molecular identification revealed that they belonged to five taxa (Table 1). Four isolates from protocorms on pine bark and two isolates from protocorms on rotten oak leaf were used for in vitro symbiotic germination. Among these six isolates, amplicon sequencing revealed corresponding sequences in only three.

**Figure 4:**
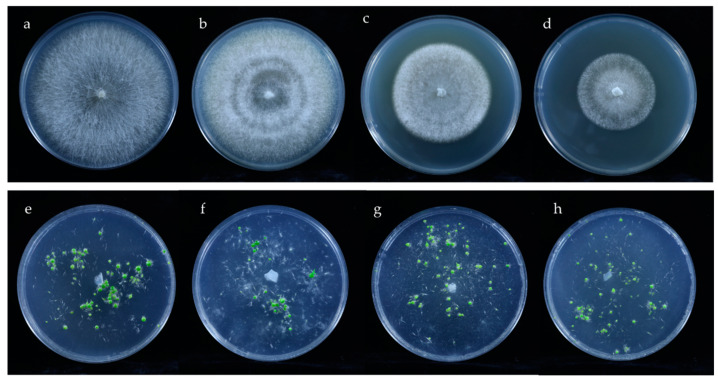
Colony morphology and in vitro symbiotic germination of *E. radicans* with germination-enhancing fungi in 90 mm Petri dishes: (**a**–**d**) colony morphology of Epi128, Epi221, Epi124, and Epi317; (**d**–**h**) in vitro symbiotic germination of seeds with fungal isolates Epi128, Epi221, Epi124, and Epi317.

**Figure 5:**
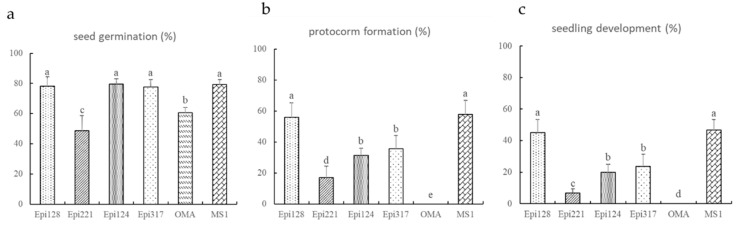
Rates of seed germination (**a**) and protocorm formation (**b**) after 30 days of germination; seedling development (**c**) after 90 days of germination.

Epi128 isolated from protocorms symbiotically germinated on rotten oak leaf was 100% identical to a *Rhizoctonia* OTU (OTU762, accession number: ON677964) (Figure S2), with a relative abundance of 18.02% in protocorms on oak leaf. Molecular identification revealed that the isolate belonged to *Ceratobasidium*. In the in vitro symbiotic germination on OMA, *Ceratobasidium* isolate Epi128 significantly promoted the germination and seedling formation of *E. radicans* (Figure 4a,e and Figure 5).

Two isolates belonging to *Tulasnellaceae* were from protocorms on pine bark and rotten oak leaf. *Peniophora incarnata* was isolated from protocorms on pine bark as Epi221. The results of in vitro symbiotic germination showed that the three isolates promoted germination of *E. radicans* comparing to the OMA control (Figure 4b–d,f–h, Figure 5 and Figure S3), while the corresponding OTU was not detected in amplicon sequencing.

The in vitro symbiotic germinated protocorms were checked microscopically for mycorrhizal colonization. Isolates Epi128 and Epi221 colonized protocorm cells of *E. radicans* and formed the typical intracellular hyphae coil of OMF (Figure S4).

As dominant fungal genera recruited from the substrates, *Fusarium* and *Trichoderma* were isolated from symbiotically germinated protocorms on pine bark. However, during in vitro symbiotic germination, the two isolates did not promote the germination, but rather inhibited the seed germination for the rapid growth of mycelia.

## 4. Discussion

This study significantly contributes to the knowledge of preferential recruitment of fungi during symbiotic germination of *E. radicans* under different microbial environments. The fungal communities in protocorms of *E. radicans* that germinated on different substrates were analyzed by barcoding analysis based on Illumina sequencing. Fungi from the symbiotic protocorms that enhanced germination were identified.

Two fungal communities were identified in symbiotic germinated protocorm of *E. radicans*. Basidiomycetous fungi were most prevalent, consistent with previous analyses that documented the associated fungi of orchids as primarily Basidiomycetes [26]. In the present study, a significant difference in the community richness of substrates did not lead to the difference in symbiotic germinated protocorms. The finding indicates that fungi recruited from two different environments might be similar in species richness and composition of fungi. In rotten oak leaf samples, Ascomycota was the most OTU-rich phylum, followed by Basidiomycota. This pattern was reversed in protocorms symbiotically germinated on rotten oak leaf. These findings indicate that there could be differences in the dominant fungal group between symbiotic germination protocorms and the germination environment. During germination, protocorms might prefer a certain group of fungi from the environment. The same patterns were found in roots of *Cypripedium calceolus*, *Neottia ovata*, and *Orchis militaris* and the soil [48].

The community structure of two types of symbiotically germinated protocorms were closer to their own substrates at the genus level. This finding indicates that *E. radicans* can recruit different fungi for seed germination according to changes in the environmental fungal community. Thus, at the genus level, protocorms might be rather opportunistic regarding the different microbial environments, with limited specificity between *E. radicans* and fungi [21,22,49]. However, at the family level, the endophytic fungal communities of the three types of protocorms clustered together, with evident similarities. This finding indicates that endophytic fungi for *E. radicans* germination on two different substrates might be with a degree of specificity at the family level.

To clarify these similarities, the shared fungi of the three types of protocorms were analyzed. In total, 12 genera belonging to nine families were shared among all three protocorm types. These can be regarded as the core fungal community for *E. radicans* germination. The functions of some core fungi in seed germination have been previously described. *Cladosporium* isolated from seed positively affected germination and plant growth in *Zea mays* and American sweetgum seedlings [50,51]. In orchids, *Cladosporium* isolated from *Dendrobium officinale* seedlings could colonize root cells of *Dendrobium* and form typical intracellular hyphae of OMF, which improved the ability of *Dendrobium* to withstand high-temperature stress and promoted the synthesis of *Dendrobium* polysaccharide [52]. Species in the large mushroom genus *Russula* are ecologically important ectomycorrhizal symbionts with forest tree species [53,54]. *Russula* have been identified from the roots of orchids, such as *Chamaegastrodia inverta* [55] and *Goodyera velutina* [56]. It is conceivable that some of the orchids were somewhat dependent on forest ectomycorrhizal fungi such as *Russula* spp.

The majority of orchids associate with basidiomycetous fungi [57]. To describe the basidiomycetous mycorrhizal community in orchids, effective broad-spectrum basidiomycete primer pairs were used. These included ITS1-OF/ITS4-OF [58], ITS1F/ITS4 [25], ITS1/ITS4-Tul [58], and ITS3/ITS4-OF [59]. This specific amplification of basidiomycetous fungi usually could not reflect the real fungal community structure in orchids. A previous study revealed a large proportion of ascomycetes fungi coincident with basidiomycetes in orchid roots using the ITS86F/ITS4 primer combination [60]. To obtain comprehensive information of fungal diversity, the universal primers ITS1F and ITS2R were gradually used in a study of orchid endophytic fungi [26], and they are commonly used to detect the endophytic fungal community of gramineous crops [9].

Ceratobasidiaceae and Sebacinales have been previously reported as dominant OMFs. They were the dominant fungal groups in symbiotic germinated protocorms on oak leaf and pine bark, respectively. Fungi in Ceratobasidiaceae were detected in both types of symbiotic germinated protocorms with two different genera (protocorms on pine bark: *Thanatephorus*; protocorms on oak leaf: *Rhizoctonia*). However, the corresponding OTU was not detected in the substrates. The reason might be that the relative abundance of fungi in the substrate was too low to be detected. After colonizing the protocorm, the biomass of the fungi increased, and the relative abundance increased significantly, reaching a detectable level in amplicon sequencing.

Although most of the endophytic fungi in *E. radicans* protocorms were not considered typical OMF, some were repeatedly observed to colonize orchid roots in previous studies. This suggests a possible germination promoting role of these fungi for their associated orchids. For instance, the genus *Clitopilus* (Entolomataceae, Agaricales, Basidiomycota), which was present in oak leaf substrate and was a dominant genus in protocorm germinated on oak leaf substrate, can establish an ectomycorrhizal association with trees in *Quercus* [61,62,63]. A recent study documented the broad host range of *C. hobsonii* and its ability to colonize roots of the phylogenetically distant tree lineages, American sweetgum (*Liquidambar styraciflua*), and poplar (*Populus* sp.) [64]. Accordingly, *Clitopilus* may have the potential to promote seed germination of orchids. A similar scenario could exist for other basidiomycetous fungal species in pine bark samples, including *Cylindrocarpon*, which has been isolated from the roots of orchid species *Arundina graminifolia* [47].

More comprehensive information about the orchid endophytic fungal community can be obtained by amplicon sequencing. However, identification of fungal function is inseparable from the isolation and identification of fungi. The fungi isolated from symbiotic germinated protocorm revealed rhizoctonia-like isolate Epi128 in Ceratobasidiaceae (*Ceratobasidium* sp. AG-A), which promotes seed germination of *E. radicans*. *Rhizoctonia* (*Ceratobasidium*) AG-6, AG-G, P, and R isolated from Orchidaceae also increase seed germination and promote protocorm growth compared to asymbiotic treatments [65].

Tulasnellaceae is a dominant mycorrhizal group in both terrestrial and epiphytic green orchids. However, amplicon sequencing did not detect Tulasnellaceae in all samples. Fungi belonging to Tulasnellaceae were isolated from symbiotic germinated protocorms. These findings may reflect the preference of primer or the small biomass of fungi leading to the failure of amplification. In vitro symbiotic germination of culturable Tulasnellaceae fungi promoted the germination of *E. radicans*. The results are consistent with previous data on the promotion of orchid germination by Tulasnellaceae [66].

In addition to previously reported dominant OMF, a fungal isolate belonging to *Peniophora incarnata* could promote seed germination of *E. radicans*. The fungal hyphae colonized the inner cells of the protocorm. White-rot basidiomycetous fungi (*Peniophora* spp.) readily produce ligninolytic enzymes [67] and were candidates for biocontrol studies of *Eutypella* canker of maple (*Eutypella parasitica*) [68]. The present study is the first report on the promoting effect of *P. incarnata* on the seed germination of orchids.

## 5. Conclusions

The community structures of endophytic fungi in protocorms germinated under different environments were close at the family level. In contrast, fungi recruited by protocorms on different substrates may be rather opportunistic at the genus level in different microbial environments. The analysis of the similarities and differences in fungal communities recruited by seed germination in different environments is critical to unveil the adaptation and utilization mechanism of the seed to different fungal communities during germination. The germination-enhancing *Peniophora incarnata*, as a white-rot basidiomycetous fungus, will have value to explain the evolution of endophytic fungi to orchid mycorrhizal fungi in the waiting room hypothesis. The method of ex situ symbiotic germination of *E. radicans* has potential value to reduce the dependence on plant tissue culture in orchid cultivation and breeding, save cost, and allow more people to participate in the orchid industry.

## Figures and Tables

**Table 1:** Molecular identification of fungi isolated from ex situ symbiotically germinated protocorms.

Isolate Number	Substrate	Closest Match in GenBank (Accession Number)	Taxonomic Affiliation	Accession Number	Identity	Corresponding OTU
Epi124	Pine bark	KM211337	Uncultured Tulasnellaceae	ON629737	99.65%	Not found
Epi221	Pine bark	MK842111	*Peniophora incarnata*	ON629738	99.83%	Not found
Epi1217	Pine bark	MN959990	*Fusarium oxysporum*	ON629747	100.00%	OTU728
Epi1417	Pine bark	MK870486	*Trichoderma* spp.	ON629749	99.66%	OTU602
Epi128	Oak leaf	KR259886	*Ceratobasidium* spp. AG-A	ON629750	98.78%	OTU762
Epi317	Oak leaf	LC480736	Uncultured Tulasnellaceae	ON629753	99.83%	Not found

## Data Availability

The data used in the study are available upon request from the corresponding author.

## Supplementary Materials

The following supporting information can be downloaded at https://www.mdpi.com/article/10.3390/microorganisms10091841/s1: Table S1. OTUs of all samples; Figure S1. Shannon rarefaction curves of the ITS reads based on OTUs at 97% sequence similarity; Figure S2. Sequence alignment between OTU762 and Epi128; Figure S3. Germination of *E. radicans* on OMA medium; Figure S4. Fungal hyphae colonized in the inner cells at the basal part (the suspensor end) of protocorms and formed the intracellular hyphae coils after in vitro symbiotic germination with Epi128 (a) and Epi221 (b); Figure S5. Hierarchical clustering tree of the samples on genus level (a) and family level (b).
