# Supplementary material for: Epidendrumradicans Fungal Community during Ex Situ Germination and Isolation of Germination-Enhancing Fungi
Source: Microorganisms. 2022 Sep 15;10(9):1841. doi: 10.3390/microorganisms10091841 (PMC9503211; doi:10.3390/microorganisms10091841)
Supplement: Supplementary file 1 [file microorganisms-10-01841-s001.zip › microorganisms-1827875-supplementary.pdf]

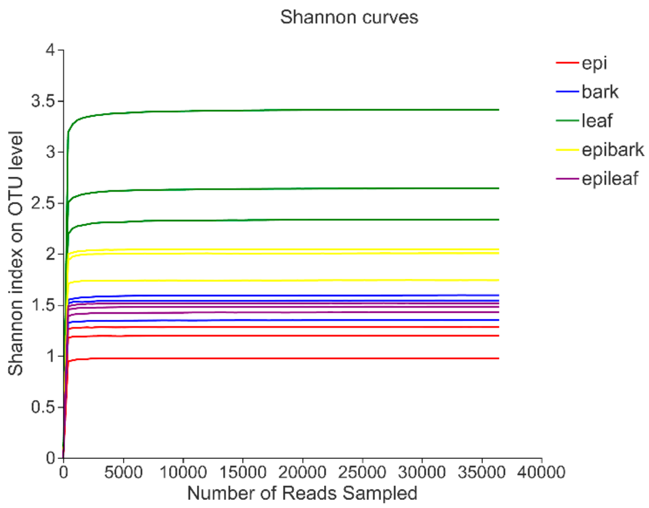

Figure S1. Shannon rarefaction curves of the ITS reads based on OTUs at 97% sequence similarity

```
OTU762      -----------------------------------------------------GAGAGCC
Epi128      GAATTCTGCAATTCACATTACTTATCGCATTTCGCTGCGTTCTTCATCGATGCGAGAGCC
                                                                 *******

OTU762      AAGAGATCCGTTGTTGAAACTTAGTTTAATGATGCGTTACATCAAGTACATTCAGTTCAA
Epi128      AAGAGATCCGTTGTTGAAACTTAGTTTAATGATGCGTTACATCAAGTACATTCAGTTCAA
            ************************************************************

OTU762      TTAAATGAGTTTGTATATGAATTTAGCAGACTGAAAGCTTTTTACGGCCTCCAGTCTCAC
Epi128      TTAAATGAGTTTGTATATGAATTTAGCAGACTGAAAGCTTTTTACGGCCTCCAGTCTCAC
            ************************************************************

OTU762      AGGTGCACAGGGGTGTGTGGATGAAAGGAGAAGGCGTGCACATGCTGCCAAAGACAGCCA
Epi128      AGGTGCACAGGGGTGTGTGGATGAAAGGAGAAGGCGTGCACATGCTGCCAAAGACAGCCA
            ************************************************************

OTU762      GCGACAACCAACCCCAAGTTCATTCAATAATGATCCTTCCGCAGGTTCACCTACGGAAAC
Epi128      GCGACAACCAACCCCAAGTTCATTCAATAATGATCCTTCCGCAGGTTCACCTACGGA---
            *********************************************************

OTU762      CTTGTTACGACTT
Epi128      -------------
```

Figure S2. Sequence alignment between OTU762 and Epi128. Asterisk represents that the nucleotide base in the two sequences at this site is identical.

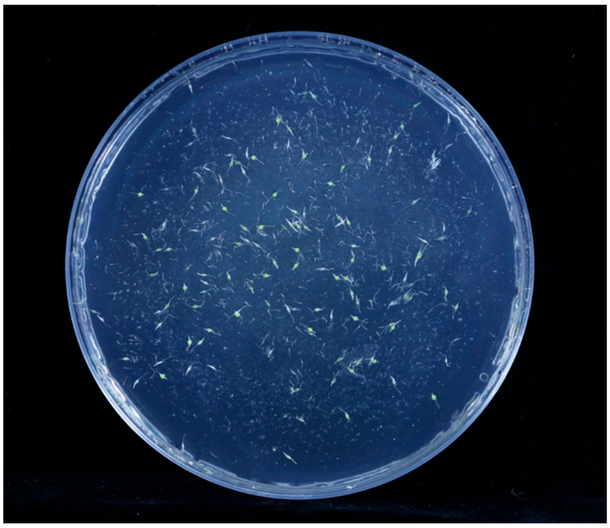

Figure S3. Germination of *E. radicans* on OMA medium in 90 mm Petri dish

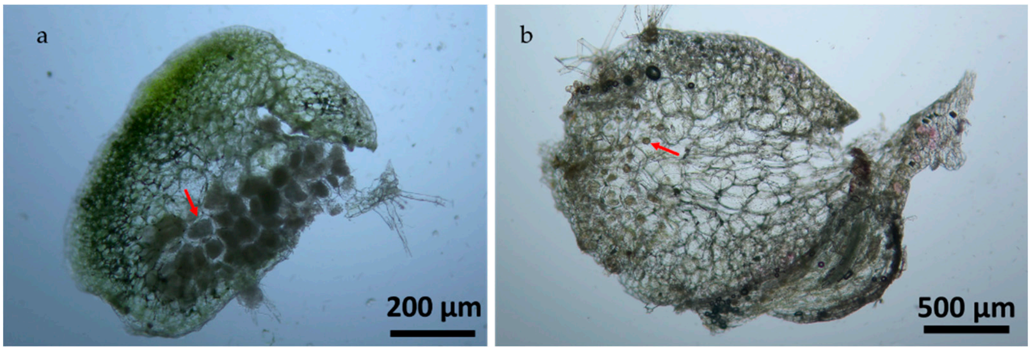

Figure S4. Fungal hyphae colonized in the inner cells at the basal part (the suspensor end) of protocorms and formed the intracellular hyphae coils after *in vitro* symbiotic germination with Epi128 (a) and Epi221 (b). The red arrow indicates the intracellular fungal peloton.

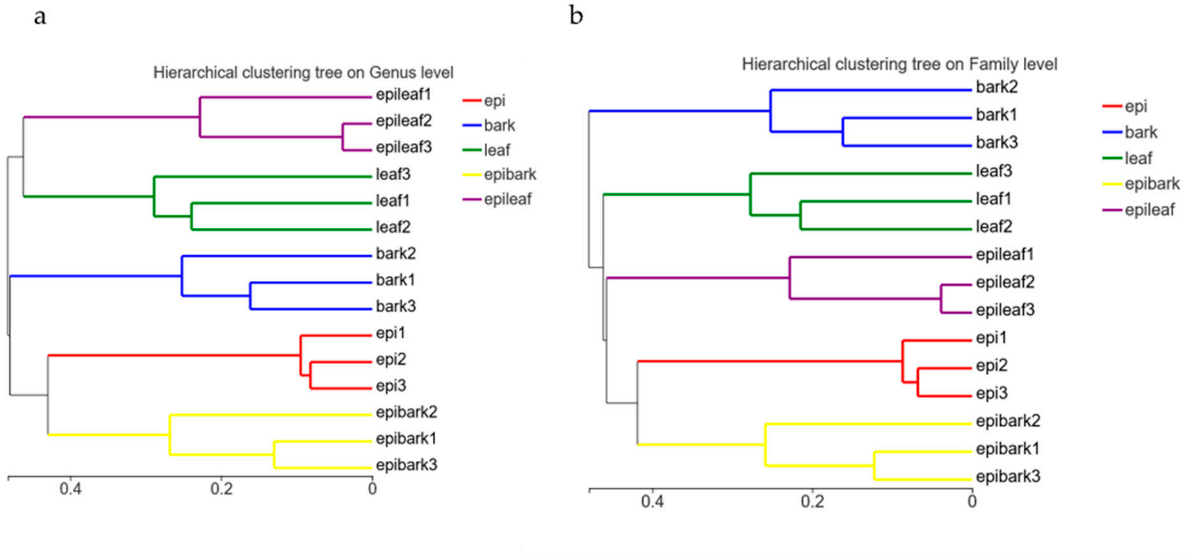

Figure S5. Hierarchical clustering tree of the samples on genus level (a) and family level (b)
